# Body composition in professional female netball players within and between seasons: a cohort study

**DOI:** 10.1186/s13102-021-00287-z

**Published:** 2021-06-04

**Authors:** Luke Hogarth, Ava Farley, Max McKenzie, Brendan Burkett, Mark McKean

**Affiliations:** 1 School of Health and Behavioural Sciences, University of the Sunshine Coast, Queensland 4556 Sippy Downs, Australia; 2 High Performance Sport, University of the Sunshine Coast, Queensland 4556 Sippy Downs, Australia

**Keywords:** Anthropometry, DXA, Bone mineral content, Team sport training, Adaptation

## Abstract

**Background:**

There is limited information on the physique attributes of female netball players from the highest playing standards and the typical body composition changes that occur with training and competition in these athletes. The purpose of this study was to examine the body composition of professional female netball players and changes that occur within and between national premier netball seasons.

**Methods:**

Dual-energy X-ray absorptiometry (DXA) assessments were conducted in 20 female netball players (age = 26.5 [4.7] years, body mass = 77.3 [9.7] kg, stature = 182.7 [9.5] cm) contracted to a Suncorp Super Netball team. Total body lean mass, fat mass, bone mass and bone mineral density were derived for 127 assessments collected over three seasons. Linear mixed effects modelling was used to examine changes in body composition measures within and between seasons.

**Results:**

Goal circle players were heavier (12.3 [3.5] kg, *p* < 0.001, *g* = 1.51) and taller (15.0 [2.7] cm, *p* < 0.001, *g* = 2.30) than midcourt players, and midcourt players had greater lean mass (3.1 [1.6] %, *p* = 0.07, *g* = 0.85) and less fat mass (-3.3 [1.7] %, *p* = 0.06, *g* = -0.84) than goal circle players when values were normalised to body mass. Players achieved increases in lean mass (2,191 [263] g, *p* < 0.01, *g* = 0.45) and decreases in fat mass (-835 [351] g, *p* = 0.09, *g* = -0.16) following a preseason preparation period. There were no changes in lean mass (-394 [295] g, *p* = 0.54, *g* = 0.07) or fat mass (102 [389] g, *p* = 0.99, *g =* 0.04) from the start to the end of the 14-week competition period.

**Conclusions:**

Professional female netball players achieve small changes in lean mass and fat mass during preseason preparation and maintain their physique over the competitive season. The results of this study can inform practitioners on the training content necessary to promote or maintain desired body composition changes in these athletes.

## Background

Netball is a team court sport that is most popular in Commonwealth countries and is one of the professional women’s sports played in Australia. Suncorp Super Netball is the national premier competition in Australia that was established in 2016 and, based on the standard of competition and number of international players, is one of the most reputable netball competitions in the world. The increased professionalism of netball in recent years has improved the standard of training and preparation practices of teams and has driven research efforts to better understand the determinants of netball performance at the highest playing standards [1–3].

Played over 15-minute quarters, netball is a fast-paced game that can be characterised by high intensity intermittent bouts of play that require a range of physical qualities [4–6]. Court positions have differing roles within game play, which has guided the optimal physical characteristics required to achieve competitive success [7–9]. There is limited research on the body composition of professional netball players from the highest playing standards or the physique traits associated with specific playing positions [7]. Many sports have established the association between physique traits and athletic performance with high levels of muscularity necessary to gain a competitive advantage [10–16]. Similarly, research in netball has shown players from higher standards have greater body mass and less body fat percentage than players from lower standards [7]. This might be explained by a relationship between muscle volume, cross-sectional area and muscular strength that is also a determinant of jumping, sprinting and change of direction performance in netball players [17–19]. Nevertheless, further research is needed to establish the physique traits of netball players from the highest playing standards and differences that exist between playing positions.

It is routine practice to monitor body composition in athletes to optimize training and dietary practices with the intent of enhancing performance outcomes [20, 21]. Body composition changes in professional netball players over consecutive seasons have been reported previously [7]. This study reported small to moderate increases in body mass and bone mineral density, and small decreases in relative fat mass over three seasons although the analysis included a different cohort of players each season. To the authors knowledge there is no published research on the typical body composition changes that occur in professional netball players within season. Highly trained athletes only show small changes in physique traits over time, especially from the start to the end of a competitive season [22–24]. Any change in body composition is likely influenced by the varied training and match loads that occur within the preseason training and competition periods.

 Information on the typical changes in body composition and other fitness traits that occur within season can inform training and dietary interventions aimed at maintaining the optimal physique traits during the competition period. However, there is limited published information on the body composition of professional female netball players, the physique traits associated with specific playing positions, or changes that occur with training and competition. This study aims to (i) examine the physique traits of professional female netball players stratified by playing position, and (ii) establish the typical changes in body composition that occur in these athletes within and between national premier netball seasons.

## Methods

### Participants

 Twenty professional female netball players volunteered to participate in this cohort study (age = 26.5 [4.7] years, body mass = 77.3 [9.7] kg, stature = 182.7 [9.5] cm). Participants included international representatives (*n* = 9), squad players (*n* = 7), and training partners (*n* = 4) contracted to the same Suncorp Super Netball team. This study was approved by the University of the Sunshine Coast Human Research Ethics Committee in accordance with the Declaration of Helsinki (A16885). The participants were informed of the benefits and risks of the investigation prior to signing an institutionally approved informed consent document to participate in the study.

### Design

Dual-energy X-ray absorptiometry (DXA) scans were conducted at four time points during Suncorp Super Netball seasons. The initial assessment (T1) occurred prior to commencement of preseason training. This typically followed a 10 to 12-week offseason where the athletes maintained an unstructured general fitness routine. The second assessment (T2) occurred three months later during the middle of preseason training that was managed by the physical performance coach. The third assessment (T3) occurred three months later in the last week of preseason training prior to round one of the competition. The fourth assessment (T4) occurred 14 weeks later during the last round of the home-and-away competition prior to the finals. Table 1 shows an overview of the training content prescribed during the season.

**Table 1:** General overview of weekly training content in professional netball players during the preseason preparation and competition periods of the Suncorp Super Netball season

Period	Objectives	Mode	Frequency	Duration^c^	Intensity
Preseason 1	Aerobic capacity	Off legs^a^	3	30–60 min	6–7 RPE
	Anaerobic capacity	On/off legs	2	30–35 min	7–8 RPE
	Straight line speed	On legs^b^	1	30 min	9 RPE
	Strength and hypertrophy	Gym	3	70–80 min	P: 3–8 reps 80–95 % 1RM; S: 6–12 reps 70–80 % 1RM
Preseason 2	Aerobic power	Off legs^a^	2	30–40 min	7–8 RPE
	Anaerobic power	On/off legs	2	30–35 min	8–9 RPE
	Speed and COD	On legs^b^	2	30 min	9–10 RPE
	Maximal strength and power	Gym	3	60–70 min	MS: 1–6 reps 90–100 % 1RM; MP: 2–6 reps 40–60 % 1RM
Competition	Speed and COD	On/off legs	2	30 min	9 RPE
	Maintenance of strength and power	Gym	2	50–60 min	MS: 3–6 reps 85–90 % 1RM; MP: 2–6 reps 40–60 % 1RM
Offseason	Aerobic capacity	On/off legs	3	90–120 min	4–6 RPE
	Strength and endurance	Gym	2	60–75 min	5–6 RPE

*RPE* rate of perceived exertion using the CR-10 scale, *P* Primary lifts, *S* Supplementary lifts, *MS* Maximal strength exercise, *MP* Muscular power exercise, *Reps* repetitions, *1RM* 1-repetition maximum

^a^off legs: conditioning using mixture of indoor cycling, rowing, cross training

^b^on legs: conditioning using mixture of track work and court training

^c^inclusive of warm up and cool down unless blocked with other forms of training. Note: Athletes undertook individualized training programs, and there were some variations in exercise selection and programming, depending on their physique, performance goals, and training experience

### Procedures

Guidance was provided in advance of the DXA scan to ensure subject presentation was standardized [25]. In order to minimise biological variability participants were required to present overnight fasted with no fluid intake and well rested (no prior physical activity) on the morning of the scan. They were asked to wear minimal fitted clothing with metal objects and jewellery removed, and clothing was checked for metal zips or studs. All participants voided their bladder prior to tests. The testing sessions commenced with stretch stature which was measured with a wall mounted stadiometer (Harpenden, Holtain Limited, Crymych, United Kingdom) to the nearest 0.1 cm using techniques previously described [26]. Body mass was measured with a calibrated scale to the nearest 0.1 kg (AND HW-200GL, A&D Australasia Pty Ltd, Thebarton, South Australia).

Scans were undertaken in the total body mode on a narrow fan beam DXA scanner (Lunar iDXA, GE Healthcare, Madison, WI) with analysis performed using GE enCORE v.13.60 software (GE Healthcare) with the Geelong reference database. The coefficient of variation for the laboratory being 0.1 %, 2.2 %, 0.6 %, 1.0 % for body mass, fat mass, lean mass, and bone mass, respectively. The DXA was calibrated with phantoms as per the manufacturer’s guidelines each day before measurements were taken. Scans were conducted by the same Queensland Radiation Health licensed technician using the standard thickness mode as determined by the auto scan feature in the software and all safety protocols as per the Institution’s Radiation Safety Protection Plan were adhered to.

The protocol emphasized consistent positioning of participants on the DXA scanning bed [27]. Two Velcro straps were used to minimise movement during the scan as well as provide a consistent body position for subsequent scans. One strap was secured around the ankles above the foot positioning pad and the other strap was secured around the trunk at the level of the mid forearms [27]. Scans were analyzed automatically by the DXA software, but all regions of interest were reconfirmed before analysis.

### Statistical analysis

Statistics were performed using R version 3.6.1. Descriptive statistics were calculated for goal circle players (goalkeeper, goal defence, goal shooter, goal attack) and midcourt players (centre, wing defence, wing attack) based on participants’ preferred playing position. Normality of distribution of data was confirmed using the Shapiro-Wilk’s test. Independent sample t-tests were used to determine differences in body composition and bone mineral density between positional groups. The mean of multiple test measures from the same participant was used for analyses to avoid pseudoreplication. Hedges’ *g* effect sizes were calculated and reported with 95 % confidence intervals. They were interpreted as negligible (≤ 0.20), small (0.21–0.50), medium (0.51–0.8) or large (> 0.8).

Linear mixed effects modelling was used to examine changes in body composition within and between consecutive seasons. Random-intercept models were trained with the time point of measurement within season included as a fixed effect. Variables identifying the athlete and playing season were included as crossed random effects. Data for participants that had a minimum of three assessments within the same season were included in this analysis (participants = 20, cases = 36, measures = 127). Random-intercept models were also trained with season included as a fixed effect, and athlete and time point included as crossed random effects, to examine changes in body composition between consecutive seasons. Data for participants that had at least three assessments each season for two or more consecutive seasons were included in this analysis (participants = 11, cases = 27, measures = 94).

Estimated marginal means were calculated to determine differences between time points within and between seasons. The Kenward-Roger method was used to derive P values with a Tukey adjustment for multiple comparisons. The mean of the estimate and the standard error were derived, and Hedges’ g effect sizes were calculated to interpret the direction and magnitude of change.

## Results

Descriptive statistics for participants stratified into goal circle and midcourt positional groups are shown in Table 2. Goal circle players were taller (15.0 [9.3–20.8] cm, *p* < 0.01) and heavier (12.3 [4.8–19.8] kg, *p* < 0.01) than midcourt players. There was a trend of goal circle players having greater fat mass (3.3 [-0.3–6.8] %, *p* = 0.07) and less lean mass (-3.1 [-6.6–0.4] %, *p* = 0.08) than midcourt players when values were normalized to body mass or stature.

**Table 2:** Body composition and bone mineral density of netball players stratified by positional group

	Midcourt players		Goal circle players			
Variable	Mean [SD]	Range	Mean [SD]	Range	Hedges’ *g* [95 % CI]	*P* value
	*n* = 7		*n* = 13			
Age, y	27.8 [3.3]	23.4–32	25.8 [5.3]	18.2–34.7	0.40 [-0.55, 1.36]	0.32
Stature, cm	172.9 [5.2]	167.6–180	187.9 [6.7]	177.5–199	-2.30 [-3.50, -1.11]	< 0.01
Body mass, kg	69.4 [7.0]	62.5–81.7	81.6 [8.2]	67.9–98.1	-1.51 [-2.56, -0.45]	< 0.01
Bone mass, g	3094 [315]	2681–3510	3475 [298]	3128–3992	-1.20 [-2.22, -0.18]	0.02
Scaled bone mass, kg∙m^− 2^	1.03 [0.07]	0.95–1.12	0.99 [0.1]	0.83–1.13	0.47 [-0.48, 1.43]	0.26
Relative bone mass, %	4.4 [0.3]	4.1–5	4.3 [0.4]	3.4–4.9	0.38 [-0.57, 1.33]	0.37
Bone mineral density, g∙cm^2^	1.42 [0.08]	1.34–1.51	1.43 [0.09]	1.32–1.63	-0.12 [-1.06, 0.83]	0.78
Lean mass, g	51,758 [4856]	45,325–59,103	57,953 [3636]	51,727–61,807	-1.45 [-2.50, -0.40]	0.01
Scaled lean mass, kg∙m^− 2^	17.28 [0.99]	16.1–19.1	16.43 [1.11]	14.93–18.5	0.76 [-0.22, 1.73]	0.10
Relative lean mass, %	74.4 [3.3]	71.4–80.8	71.3 [3.6]	63.8–78.2	0.85 [-0.14, 1.83]	0.07
Fat mass, g	14,848 [3181]	10,076–19,626	20,212 [4988]	11,628–31,696	-1.15 [-2.16, -0.14]	< 0.01
Scaled fat mass, kg∙m^− 2^	4.95 [0.95]	3.32–6.34	5.72 [1.38]	3.5–8.3	-0.59 [-1.55, 0.37]	0.16
Relative fat mass, %	21.2 [3.3]	14.8–23.9	24.5 [4]	17–32.8	-0.84 [-1.82, 0.14]	0.06

Hedges’ *g* effect sizes can be interpreted as negligible (≤ 0.20), small (0.21–0.50), medium (0.51–0.8) and large (> 0.8)

*SD* Standard deviation, *CI* Upper and lower confidence interval

There was an effect of time point within season on total bone mass (χ^2^ = 15.3, df = 3, *p* < 0.01), total bone mineral density (χ^2^ = 20.4, df = 3, *p* < 0.01), and total lean mass (χ^2^ = 76.2, df = 3, *p* < 0.01). The increase in total bone mass was negligible and occurred mostly from (T1) the start of preseason to (T3) the end of preseason (28.5 [9.4] g, *p* = 0.02, *g* = 0.09 [0.04, 0.14]). The increase in bone mineral density was also negligible but occurred mostly from (T2) the middle of preseason to (T4) the end of the competitive season (0.016 [0.004] g∙cm^− 2^, *p* < 0.01, *g* = 0.15 [0.07, 0.22]).

**Fig. 1:**
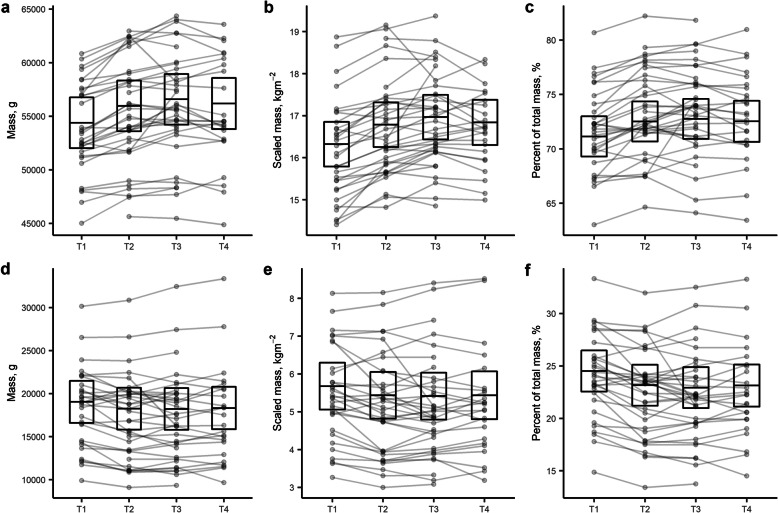
Changes in (**a**) total lean mass, (**b**) scaled lean mass, (**c**) lean mass expressed as a percentage of total mass, (**d**) total fat mass, (**e**) scaled fat mass, and (**f**) fat mass expressed as a percentage of total mass in netball players (*n* = 20) within season. Time points T1, T2, T3 and T4 refer to the start of preseason training, middle of preseason training, end of preseason training and end of competitive season, respectively. Crossbars show the mean estimate with 95 % confidence intervals and lines identify individual cases (participant and season)

There was a small increase in total lean mass from (T1) the start of preseason to (T3) the end of preseason (2,191 [263] g, *p* < 0.01, *g* = 0.45 [0.31, 0.60]). Most of the increase in lean mass occurred following the first preseason block (1,578 [263] g, *p* < 0.01, *g* = 0.32 [0.23, 0.41]) compared to the second preseason block (613 [251] g, *p* = 0.075, *g* = 0.12 [0.02, 0.23]) (Fig. 1). The cohort maintained their total lean mass from (T3) the end of preseason to (T4) the end of the competitive season (-394 [295] g, *p* = 0.54, *g* = -0.07 [-0.16, 0.02]).

There was no effect of time point within season on total fat mass (χ^2^ = 7.4, df = 3, *p* = 0.06) or scaled fat mass (χ^2^ = 7.6, df = 3, *p* = 0.055) within season. However, there was a trend showing the participant cohort achieved negligible to small decreases in absolute fat mass (-835 [351] g, *p* = 0.09, *g* = -0.16 [-0.31, -0.01]) and scaled fat mass (-0.263 [0.11] kg∙m-2, *p* = 0.08, *g* = -0.20 [-0.37, -0.02]) from (T1) the start of preseason to (T3) the end of preseason (Fig. 1). There was an effect of time point within season on fat mass expressed relative to body mass (χ^2^ = 21.4, df = 3, *p* < 0.01). The participant cohort achieved a small decrease in relative fat mass from (T1) the start of preseason to (T3) the end of preseason (-1.6 [0.4] %, *p* < 0.01, *g* = -0.38 [-0.58, -0.18]). Most of the decrease in relative fat mass occurred following the first preseason training block (-1.3 [0.4] %, *p* < 0.01, *g* = -0.32 [-0.45, -0.20]) coinciding with the largest change in fat mass and lean mass.

There was an effect of season on total bone mass (χ^2^ = 14.8, df = 3, *p* < 0.01) and total bone mineral density (χ^2^ = 15.8, df = 3, *p* < 0.01) in the subset of participants that underwent body composition assessments during at least two consecutive seasons. The largest increases in total bone mass (27 [10] g, *p* = 0.03, *g* = 0.08 [0.01, 0.16]) and bone mineral density (0.011 [0.004] g∙cm^− 2^, *p* = 0.01, *g* = 0.14 [0.02, 0.25]) over consecutive seasons were negligible and comparable to those changes reported within season.

## Discussion

This study details the physique attributes of professional female netball players and the changes in body composition that occur within and between seasons. The main findings were: (i) goal circle players are taller and heavier than midcourt players, and midcourt players have greater lean mass and less fat mass normalized to body mass and stature than goal circle players; (ii) players achieved small increases in lean mass and small decreases in fat mass during preseason preparation; and (iii) there were no changes in lean mass or fat mass in the cohort from the start to the end of the competition period.

Previous research has suggested that netball players at the highest playing standard are taller and heavier than their peers at lower playing standards [7]. However, even within a professional netball team there is considerable variation in the stature and body mass of players that is explained by their playing position (Table 2). In agreement with research in state-level netball, goal circle players were found to be taller than midcourt players [9]. The importance of gaining possession of circle feeds, shooting, and contesting for rebounds suggests taller goal circle players might have an advantage over their shorter opponents. Comparatively, the minimal restrictions on court movement for midcourt players places greater emphasis on lateral accelerations and changes of direction to gain possession and move the ball towards the goal circle [8]. Being taller might be less of an advantage for midcourt players, and instead these players tend to have higher lean mass and lower fat mass relative to their body mass and stature compared to goal circle players. Reducing fat mass and increasing muscle mass is likely a training objective of both midcourt and goal circle players given the importance of relative muscular strength to court-based jumping, sprinting and change of direction performance [18, 19]. However, lower fat mass might have greater importance to midcourt players because of the higher frequency and duration of efforts where they’re required to overcome their body’s inertia and the greater distance that they cover on court compared to goal circles players [2, 8]. These results suggest that playing position should be considered in benchmarking the physique traits of netball players for the purposes of talent identification and training program design.

There were changes in body composition within season in the cohort, with the largest changes occurring over the preseason preparation period. The most noticeable change following preseason training was a small increase in lean mass (2.19 [0.26] kg, *g* = 0.45), while there was only a negligible decrease in fat mass (-0.84 [0.35] kg, *g* = -0.16). Collegiate female basketball players have shown similar changes in lean mass (1.5 kg) and fat mass (-1.3 kg) over a preseason although differences in age, initial fitness, preseason duration and training are likely to account for varied responses between cohorts [28]. Regardless, the changes in lean mass and fat mass in the cohort were larger than the typical error values that have been reported in a mixed-sex cohort using comparable test methods [27]. These results suggest that the small changes in body composition in these athletes following training can be detected using DXA.

There were different time-courses in the change in fat mass, lean mass and bone mass because of the altered mode, frequency, volume and intensity of training throughout the preparation and competition phases of the season. The greater focus on increasing muscular hypertrophy and aerobic exercise capacity in the first training block resulted in the largest change in lean mass and fat mass (Fig. 1). These body composition changes coincide with the largest improvement in absolute and relative muscular strength during preseason training in this cohort [19]. The successive increase in lean mass following the second preseason training block suggests maximal muscular strength training also induced a hormonal response associated with increased muscle mass, albeit not to the same extent as the first training block. Collectively, these results demonstrate professional female netball players can achieve small body composition changes associated with improved athletic performance in response to a preseason preparation period.

Participants were able to maintain the increased lean mass that they achieved during the preparation period throughout the 14-week competition. Netball athletes have altered hormonal, physical and subjective well-being states following match-play that dictates the prescribed training during the competitive season to promote recovery between matches played six to eight days apart [1, 3]. Modified in-season training loads in other team sports are associated with decreases in lean mass and increases in fat mass coinciding with reduced muscle strength, endurance, speed and agility throughout the competitive season [29]. The absence of an in-season detraining effect in this study’s participant cohort might be explained by the short competition period or shorter time-course of altered hormonal and physical states following match-play. Regardless, the increase in bone mineral density in the participant cohort at the end of the competitive season suggests netball players are exposed to a large amount of mechanical loading in training and competition that induces an adaptive bone response [30]. This finding is like research in collision and court-based team sport and supports to some extent the prescription of modified in-season training loads to promote recovery between competition [29, 31].

Participants that underwent body composition assessments over multiple seasons did not report successive increases in lean mass or decreases in fat mass. These participants might have shown a detraining effect during the offseason period despite performing an unstructured and non-prescribed fitness routine. Previous research in professional netball has shown the group mean of lean mass and fat mass in the playing squad to change over consecutive seasons, although the authors noted that there were player changes to the team each year that limits the validity of these comparisons [7]. Other research has reported female athletes to show successive increases in lean mass over three years in collegiate volleyball and swimming, but not in collegiate basketball, soccer or track [32]. Longitudinal changes in body composition in athletes over consecutive seasons is likely to be multi-factorial, being influenced by factors such as biological and training age, relative importance of physique attributes to sport performance, and scheduling and periodisation of training and competition. In this study, most players with measures over consecutive seasons were international representatives and so it is possible that the detraining effect is explained by modified training around their representative duties or that these players only show small changes in body composition within and between seasons because of their training history. If maintaining lean mass and fat mass over the course of the offseason period is an objective, then professional netball players might be required to undertake a targeted training regime to avoid detraining prior to the start of preseason preparation period.

There were negligible increases in bone mass and bone mineral density between seasons in the cohort that were comparable to the increases observed within season following the competition period. Similar changes in bone mass and bone mineral density have been shown for collegiate female basketball and volleyball players [31]. These results indicate that highly trained female netballers can increase and maintain bone mineral density with adequate mechanical loading well after puberty when skeletal bone is most responsive, albeit by only negligible amounts [30]. It is interesting to note that the bone mineral density of the participant cohort was higher than values reported for male rugby league players [29], and collegiate female basketball and volleyball players [31] emphasising the regular, high-strain mechanical loading that these athletes experience in training and competition [30, 33]. Monitoring the development and maintenance of bone in netball athletes, particularly on return from extended rest due to injury, might be important given the decline in bone mineral density with periods of inactivity and the incidence of ankle and foot fractures in netball [34–36].

The limitations of this study should be addressed. The sample size of the participant cohort was small and limited to a single team that limits the generalisability of results to other cohorts. The participant cohort also consisted of athletes at various stages of their career, and the small sample size precluded the analysis of body composition based on training age or playing status. Future research that is conducted across multiple professional teams will improve understanding of physique attributes in professional female netball players. Such research can establish the physique attributes of players stratified by specific playing position rather than broad positional grouping, and by training age or playing status to better guide athlete development practices in netball.

## Conclusions

This study examined the physique traits of professional female netball players and the typical changes in body composition and bone mineral density that occur within and between national premier netball seasons. There were differences in physique attributes between positional groups. Goal circle players were taller and heavier than midcourt players, and midcourt players had greater lean mass and less fat mass relative to their body size than goal circle players. Netball players achieved increases in lean mass and decreases in fat mass during preseason preparation and maintained their physique over the competition period. There were no body composition changes between consecutive seasons, except for negligible increases in bone mass and bone mineral density. These results provide practitioners with useful information on the training content necessary to promote or maintain body composition changes within and between national premier netball seasons.

## Data Availability

The datasets generated and/or analysed during the current study are not publicly available because the use of data in this way was not outlined in the approved ethics application.

## Abbreviation

DXA: Dual energy X-ray absorptiometry
